# Carbon Dioxide Adsorption in Betulin-Based Micro- and Macroporous Polyurethanes

**DOI:** 10.1002/open.201200045

**Published:** 2013-01-18

**Authors:** Jekaterina Jeromenok, Winfried Böhlmann, Christian Jäger, Jens Weber

**Affiliations:** [a] Department of Colloid Chemistry, Max Planck Institute of Colloids and Interfaces, Science Park Golm14424 Potsdam (Germany) E-mail: jens.weber@mpikg.mpg.de; [b] University of Leipzig, Faculty of Physics and GeosciencesLinnéstr. 5, 04103 Leipzig (Germany); [c] BAM Federal Institute for Materials Research and TestingDivision 1.3, Richard Willstaetter Str. 11, 12489 Berlin (Germany)

**Keywords:** carbon dioxide, gas adsorption, microporous polymers, renewable resources, solid state NMR spectroscopy

Microporous polymers, that is polymers having pores of molecular size (D<2 nm), attracted a lot of interest during the last few years.^1^–^4^ They are promising materials for gas storage or separation but could also find use in heterogeneous catalysis. Recently, the separation of the green house gas CO_2_ evoked large interest in the scientific community. Among other microporous materials, such as activated carbon,^5^–^7^ metal-organic frameworks,^8^–^10^ or zeolites,^11^ nanoporous polymers have been suggested as adsorbents,^12^–^17^ as they can combine large surface areas together with the well-known processability of common polymers. Within this contribution, we present microporous polyurethane with good CO_2_ over N_2_ selectivity. The state of CO_2_ adsorbed in the micropores is analyzed by NMR spectroscopy in order to get a better picture of the gas–polymer interactions, which have not been subject to intense investigations so far. In combination with the analysis of the adsorption data, this allows a statement on the underlying basics of the high selectivity.

One of the various ways to synthesize microporous polymers starts from the concept of intrinsic microporosity. Using stiff and contorted molecules, it is possible to prevent dense packing of the polymer chains, leaving an extraordinary high free volume behind. If the free volume is connected and can be accessed from the environment, the polymer is considered to be intrinsically microporous.^18^, ^19^ Recently, we developed microporous polyesters with promising CO_2_/N_2_ selectivity based on betulin.^20^ Betulin, a triterpene that can be extracted from birch bark in up to 30 % yield with respect to the dry weight of the bark, can be used as a building block for microporous polymers. Two hydroxy groups point out of the otherwise stiff hydrocarbon plane, thus introducing frustrated packing (=high free volume) upon polymerization. Consequently, we envisaged the synthesis of microporous polyurethanes as a next step, making use of the diol nature of betulin. Polyurethanes based on betulin have already been described in the 1980s by Russian and Finnish researchers, but no investigations were performed on the porosity of the products.^21^

Here, we targeted the synthesis of microporous networks, as these can typically result in higher porosities. Hence, a commercial triisocyanate (20 % triphenylmethane triisocyanate in ethyl acetate, Desmodur® RE, Bayer AG) was used as a crosslinker in an A_3_-B_2_ polyaddition reaction to prepare microporous polymer networks (see Scheme 1). Betulin was extracted from birch bark and recrystallized from ethanol before use.

**Scheme 1:**
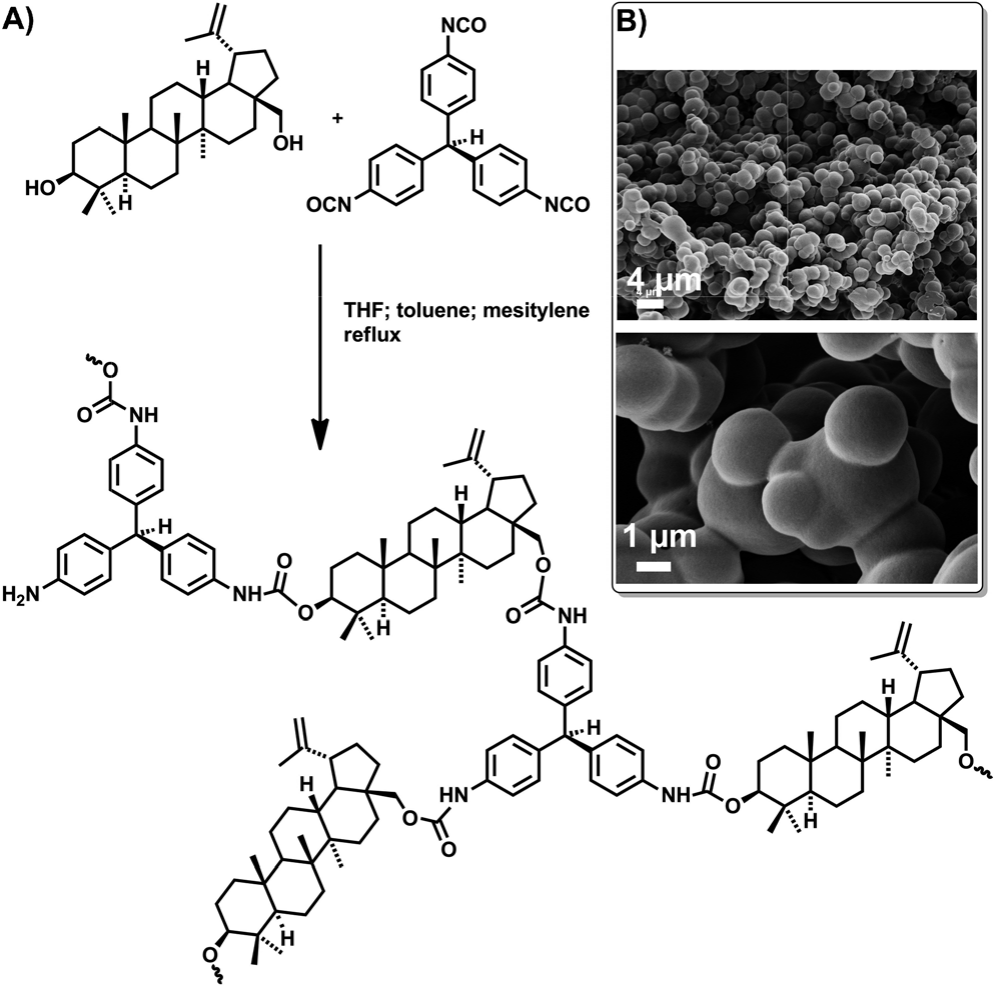
A) Chemical structures of betulin and triphenylmethane triisocyanate and the corresponding polyurethane network. B) SEM micrographs of the morphology of the resulting networks.

The reaction was performed at boiling temperature in various solvents such as tetrahydrofuran (THF), toluene or mesitylene. The polymer precipitated out of solution as the reaction proceeded, typically as interconnected microglobules of less than 10 μm diameter (see Scheme 1 B). Such morphology is typically observed and used in monolithic polymer materials used for separation applications, as it allows fast mass transfer.

Dry solvents can be used in order to prevent potential foaming due to the formation of CO_2_ and significant amounts of urea linkages. Foaming could interfere with the intrinsic porosity introduced by the betulin stereochemistry as discussed earlier. However, no strong difference was observed between the outcomes of reactions performed in dry or analytical grade solvents. Nevertheless, dry toluene was used to prepare the sample named Bet-PUR-1, which will be discussed in detail in the following.

The chemical identity of the networks was confirmed by FTIR and solid-state NMR spectroscopy. FTIR indicated the successful formation of the urethane linkage as evidenced by the reduction of hydroxy vibrational modes at 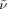
>3000 cm^−1^ and the rise of a broad carbonyl band at 1702 cm^−1^ and an amide NH-bending mode at 1592 cm^−1^. Solid-state ^1^H-^13^C cross-polarization magnetic angle spinning (CPMAS) spectroscopy can also confirm the urethane formation (Figure 1 B). The carbonyl C atom causes a peak at a chemical shift of *δ*=154 ppm. The accompanying shoulder of this peak can be attributed to the tertiary C atom of the isobutylene unit of betulin. The peaks at *δ* values of ∼137, ∼130 and ∼119 ppm can be attributed to the aromatic C atoms of the Desmodur® RE unit, while the peak at *δ*=110 ppm is also due to the isobutylene unit. The peaks at *δ* values of ∼82 ppm and ∼63 ppm can be attributed to the oxygen bearing C atoms of betulin. The peaks at lower chemical shift are due to the aliphatic betulin backbone and the tertiary C atom of the Desmodur® RE unit, which however cannot be distinguished within the spectrum.

**Figure 1:**
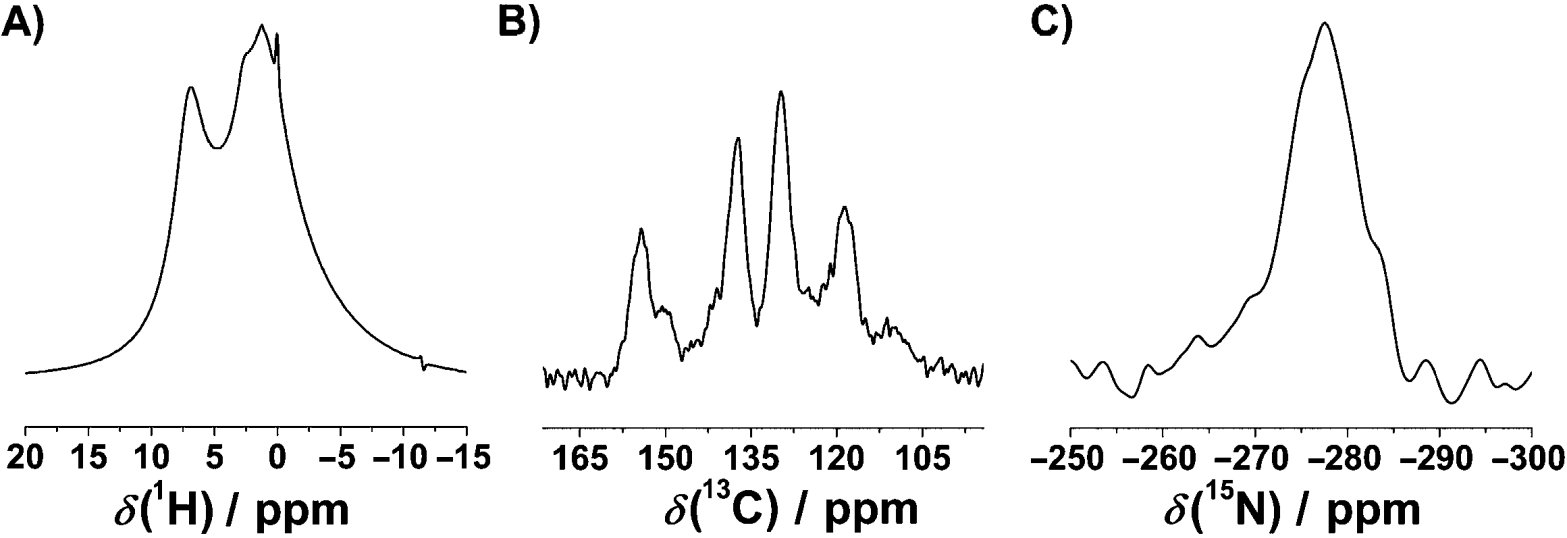
A) Solid-state ^1^H NMR spectrum, B) solid-state ^1^H-^13^C CPMAS NMR spectrum and D) solid-state ^1^H-^15^N CPMAS NMR spectrum of a botulin-based polyurethane network (Bet-PUR-1).

Thermal analysis of the networks under an N_2_ atmosphere showed a two-step decomposition. At ∼110 °C, solvent loss is observed. Decomposition begins at 350 °C with a very sharp mass loss of 42 % followed by a second broad decomposition step at 590 °C. Differential scanning calorimetry (DSC) of the freeze-dried network did not show any phase transition or glass point within a temperature range from room temperature up to 125 °C, indicating a stable operation range for adsorption applications.

The porosity of the formed polymer networks was investigated by gas adsorption/desorption measurements using N_2_ and CO_2_ as adsorbates.^22^, ^23^ N_2_ adsorption at 77.3 K could not reveal any pronounced microporosity of the polymer networks (*S*_BET_∼5 m^2^ g^−1^), irrespective of the applied drying method (freeze-drying or evaporative drying). The absence of measurable microporosity at 77.3 K could be due to partial pore closure as a consequence of hydrogen bonding but also due to restricted access of N_2_ at 77.3 K into the narrow pores because of diffusional problems.^24^ It was shown in previous studies that intermolecular interactions can indeed lead to reduction of pore sizes by elastic deformations.^23^ In the present case, the asymmetric shape of the peak originating from the urethane nitrogen as observed in the ^1^H-^15^N CPMAS NMR spectrum indicates that there are indeed additional interactions. The peak shows at least two shoulders, which could indicate the presence of hydrogen-bonded structures. This would also be supported by the rather broad C=O and N–H peaks within the IR spectra, which could be due to overlapping of “free” and hydrogen-bonded moieties.^25^ However, at the current state, we cannot exclude the presence of urea or biuret functionalities. As dry solvents have been used, we are however confident that they would be small in number and not solely be responsible for the broadened and anisotropic peaks observed by spectroscopy.

Additional porosity analysis was undertaken by CO_2_ adsorption at 273 and 283 K as well as N_2_ adsorption at 273 K. This set of experiments allows to detect microporosity and to calculate apparent gas selectivities and CO_2_ adsorption strength. It was shown previously that CO_2_ can enter very small pores in polymers or carbons that otherwise could not be detected.^20^, ^24^, ^26^

The CO_2_ uptake of Bet-PUR-1 at 273 K and 1 bar was around 1.26 mmol g^−1^ (5.5 wt %; see Figure 2 A) indicating moderate microporosity. Using models developed for carbons,^27^ it is possible to determine the specific surface area (S∼300 m^2^ g^−1^) and the pore size distribution (PSD, see the Supporting Information). The majority of pores seem to have pore sizes between 0.5 and 1 nm. Such small pore sizes can be interesting for kinetic gas separations. CO_2_ is known to have a smaller kinetic diameter than N_2_ and a separation based on kinetic restrictions can be imagined. The N_2_ uptake at 273 K and 1 bar was 0.065 mmol g^−1^. The adsorption isotherms could be fitted using a dual or single-site Langmuir model in the case of CO_2_, while a simple Langmuir fit was sufficient to fit the N_2_ isotherm (see the Supporting Information for full fit details). Based on the fit data, it is possible to calculate the CO_2_/N_2_ selectivity using the ideal adsorbed solution theory (IAST) approach. A selectivity of ∼130 can be calculated for a gas composition of 15 % CO_2_ and 85 % N_2_ at 273 K and 800 mm Hg. This is a high value, which must however be placed into relation to the capacity, which is lower than that of other microporous polymers, following the trade-off relation between capacity and selectivity.^28^ Nevertheless, the preliminary results are encouraging for further study of the gas separation properties of betulin-based polyurethanes.

**Figure 2:**
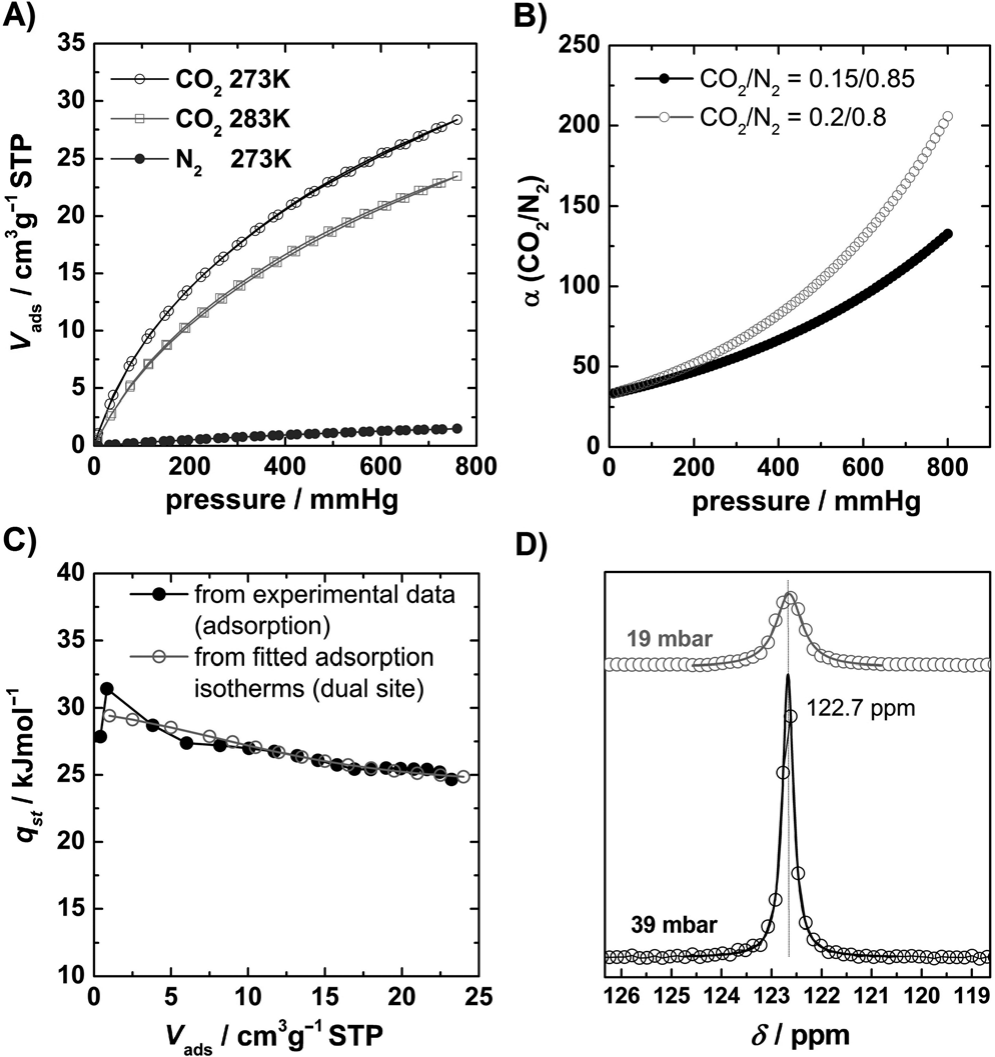
A) CO_2_ (273 and 283 K) and N_2_ (273 K) adsorption isotherms on Bet-PUR-1. B) IAST prediction of the CO_2_/N_2_ selectivity (273 K) for a 0.2:0:8 and a 0.15:0.85 gas composition. C) Isosteric heat of adsorption *q*_st_, calculated directly by the AS1Win software and calculated based on the fitted isotherms. D) ^13^C NMR spectra of ^13^C-CO_2_ confined within Bet-PUR-1.

An advantage amongst other approaches towards microporous polymers lies in the fact that the engineering of polyurethanes into usable morphologies (e.g., monolithic foams) is well-developed, which could support the manufacturing of prototypes for real separation applications.

Another question, which needs to be tackled, is related to the nature of adsorbed CO_2_. There is an ever increasing number of microporous polymeric materials, which shows no N_2_ uptake at 77.3 K but CO_2_ uptake at 273 K. This brings up the question, whether CO_2_ is indeed physisorbed into micropores or if it undergoes chemical interactions leading to the observed uptake. In the present case, free amines could interact with CO_2_, and a strong interaction could also be imagined between CO_2_ and the amide N–H. A first way to screen potential interactions is the calculation of the isosteric heat of CO_2_ adsorption (*q*_st_, Figure 2 C). These can be derived directly from the commercialized software, but also from the fitted isotherms (Clausius-Clapeyron approach). Both data sets agree well, and values of ∼30 kJ mol^−1^ are calculated for zero loading. These decrease to ∼25 kJ mol^−1^ at higher loadings, indicative of pure physisorption of CO_2_ within the micropores of the betulin-based polyurethane. The slightly higher heat of adsorption at low coverage can be due to pore size effects; that is, very small pores give rise to increased wall-adsorbate interactions and/or to the presence of polar sites (urethane linkages) that also have stronger interactions with CO_2_. To further characterize the state of CO_2_ adsorbed into small pores, solid-state ^13^C NMR spectra of ^13^C-CO_2_ confined to the pores were recorded (Figure 2 D). ^13^C-CO_2_ was loaded into an NMR tube containing Bet-PUR-1, and the tube was sealed. Low pressures (<50 mbar) were used to gain information about the low coverage region. The spectra show isotropic peaks at a position of 122.7 ppm, which is comparable to values found for microporous activated carbon (118–123 ppm).^29^ The value is indicative of CO_2_, which is confined but still can undergo isotropic rotations, that is purely physisorbed CO_2_. Studies on CO_2_ confined in extremely narrow micropores (activated carbon fibres, width<0.42 nm) showed that a second peak originates at lower chemical shift, which is extremely broad as a consequence of restricted uniaxial rotation. Such a peak was not visible in our case, which can be interpreted as the absence of extremely small pores. Hence, the higher heat of adsorption at low coverage can be attributed to the polar urethane moieties, which are preferential adsorption sites to CO_2_ compared to the unipolar aliphatic betulin backbone.

In summary, an in-depth study on the characterization of betulin-based polyurethanes was presented. Betulin, which is a renewable resource and not in conflict with food production, can be used as a structure-directing monomer in an A_2_+B_3_ synthesis. Reaction with a commercial isocyanate results in a microporous polymer containing ∼64 wt % renewable material. The synthesis can be conducted in a variety of solvents, typically resulting in a structure of interconnected microglobules. Such a structure is commonly found in macroporous polymeric monoliths.^30^ Indeed, using THF as solvent, a monolithic structure could be obtained when the reaction was performed without stirring. The monolith ruptured upon conventional drying (see the Supporting Information), which is a common problem of monolithic materials and could most probably be prevented by supercritical drying. Nevertheless, this is encouraging for further engineering work on this type of polymers. The adsorption studies revealed a promising CO_2_/N_2_ gas selectivity based on the analysis of single gas adsorption isotherms. The overall capacity is not very high compared to other systems, but this could be compensated by the better processability. No binders are potentially required to form monoliths, which is advantageous compared to powder processing. The selectivity could be a result of rather small pores (kinetic separation effect),^11^ as no specific interactions could be evidenced by in situ NMR spectroscopy. The presence of the urethane linkages resulted presumably in the formation of hydrogen bonds, which are known to reduce pore sizes.

## Experimental Section

**General**: Betulin was obtained from extraction of birch bark (*betula pendula*) with CH_2_Cl_2_, followed by recrystallization from EtOH as described earlier.^20^

Elemental analyses were done with a varioMicro elemental analysis instrument (Elementar Analysensysteme, Hanau, Germany). Thermogravimetric analyses were performed in synthetic air atmosphere with a NETZSCH TG209 F1 instrument (Selb, Germany) at a heating rate of 10 K min^−1^. Differential scanning calorimetry (DSC) was performed using a Mettler-Toledo instrument under N_2_ atmosphere and a heating rate of 10 K min^−1^. Fourier transform infrared spectra (FTIR) were collected using a BIORAD FTS 6000 FTIR spectrometer under attenuated total reflection (ATR) conditions. Scanning electron microscopy (SEM) measurements were carried out using a LEO 1550-Gemini electron microscope (acceleration voltage: 3 kV). For SEM measurements the samples were coated with a thin gold layer (approx. 2 nm).

**Gas sorption**: Nitrogen sorption experiments were conducted at 77.3 K using Quadrasorb and Autosorb 1-MP from Quantachrome Instruments (Boynton Beach, FL, USA). Data evaluation was done by means of different methods in the Quantachrome programs AS1win and QuadraWin. CO_2_ and N_2_ at 273 K and 283 K were done using Autosorb 1-MP from Quantachrome Instruments. Isosteric heats of adsorption were calculated using the AS1Win software provided by Quantachrome Instruments as well as from fits of the isotherms. Before all adsorption experiments, the samples were degassed overnight at 353 K under dynamic vacuum. High purity gases were used for all measurements.

**NMR spectroscopy**: Solid state ^13^C magic angle spinning (MAS) NMR measurements under ^13^CO_2_ atmosphere were performed on a Bruker Avance 400 spectrometer at a resonance frequency of 100.61 MHz and a spinning frequency of 10 kHz. Adamantane was used as external standard (signals at 28.1 and 37.5 ppm). Solid-state NMR experiments were performed on a BRUKER Avance 400 spectrometer (9.4 T). Experiments were carried out at RT using a 2.5 mm MAS sample BRUKER double resonance probe for solid-state ^1^H and ^13^C NMR. ^1^H MAS NMR spectra (400.2 MHz) were run with rotor-synchronized echo detection for suppressing probe background signals using a MAS frequency of 25 kHz. ^1^H 90° pulse lengths of 3.3 μs were used with repetition times of 3 s, and 16 scans were accumulated. ^1^H chemical shifts were referenced versus trimethylsilane (TMS) using adamantane as external secondary standard (*δ*=1.78 ppm). ^1^H-^13^C cross polarization (CP)MAS experiments were carried out using the same 2.5 mm MAS probe, but the sample spinning frequency was 12.5 kHz. Spectra were recorded using a ^1^H 90° pulse length of 3.3 μs, a contact time of 2 ms and a repetition time of 3 s. The ^13^C spin lock field was held constant while the ^1^H spin lock field was ramped down to 50 % of its initial value. 16k scans were accumulated, and high-power proton decoupling was carried out with a 15° two pulse phase modulation (TPPM) sequence.^31^ Similarly, ^1^H-^15^N CPMAS spectra were acquired using a BRUKER 7 mm MAS probe for sensitivity reasons. The sample spinning frequency was 5 kHz. Spectra were recorded with a ^1^H 90° pulse length of 5 μs, a contact time of 1 ms and a repetition time of 3 s. Again, the ^15^N spin lock field was held constant while the ^1^H spin lock field was ramped down to 50 % of its initial value. 20 000 scans were accumulated with proton TPPM decoupling as described earlier. ^15^N chemical shifts (*δ*) are reported relative to CH_3_NO_2_ with NH_4_Cl as secondary standard (*δ*=−341 ppm).

**Typical synthesis of a polyurethane network (Bet-PUR-1)**: A 20 % solution of triphenylmethane triisocyanate in EtOAc (1.1 mL, 297 mg, 0.8 mmol; Desmodur® RE solution, Bayer AG) was introduced to a solution of betulin (531 mg, 1.2 mmol) in toluene (12 mL) at 110 °C. The mixture was held at reflux for 1.5 h. A brownish precipitate was formed after 15 min, gradually changing its color to violet. The precipitate was extracted with tetrahydrofuran (THF; Soxhlet) for 20 h and dried in vacuo at 60 °C to give Bet-PUR-1 as a white-pink powder (675 mg, 92 %): FTIR (ATR): 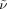
=3300 (broad, O–H), 2920 (weak, C
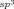

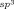
–H), 1709 (strong; C=O), 1592 (medium, N–H), 1485 (strong, C=C), 1385 (weak), 1280 (medium), 1180 (medium), 1020 (medium), 990 cm^−1^ (weak).

**Synthesis of a polyurethane monolith**: After dissolving betulin (267 mg, 0.6 mmol) in THF (3 mL) at RT in a Schlenk flask, Desmodur® RE solution (0.6 mL, approx. 0.44 mmol) was added, and the mixture was stirred shortly. The stirring bar was removed, and the reaction mixture was aged at 70 °C for 7 d. The resulting purple monolith was washed with THF and dried in vacuo at 60 °C, yielding 409 mg (95 wt %).

## Supplementary material

As a service to our authors and readers, this journal provides supporting information supplied by the authors. Such materials are peer reviewed and may be re-organized for online delivery, but are not copy-edited or typeset. Technical support issues arising from supporting information (other than missing files) should be addressed to the authors.
