# Supplementary material for: Carbon Dioxide Adsorption in Betulin-Based Micro- and Macroporous Polyurethanes
Source: ChemistryOpen. 2013 Jan 18;2(1):17–20. doi: 10.1002/open.201200045 (PMC3594587; doi:10.1002/open.201200045)
Supplement: Supplementary file 1 [file open0002-0017-SD1.pdf]

ChemistryOPEN

Supporting Information

© 2013 The Authors. Published by Wiley-VCH Verlag GmbH & Co. KGaA, Weinheim

Carbon Dioxide Adsorption in Betulin-Based Micro- and Macroporous Polyurethanes

Jekaterina Jeromenok,[a] Winfried Böhlmann,[b] Christian Jäger,[c] and Jens Weber*[a]

open_201200045_sm_miscellaneous_information.pdf

monolithic structure:

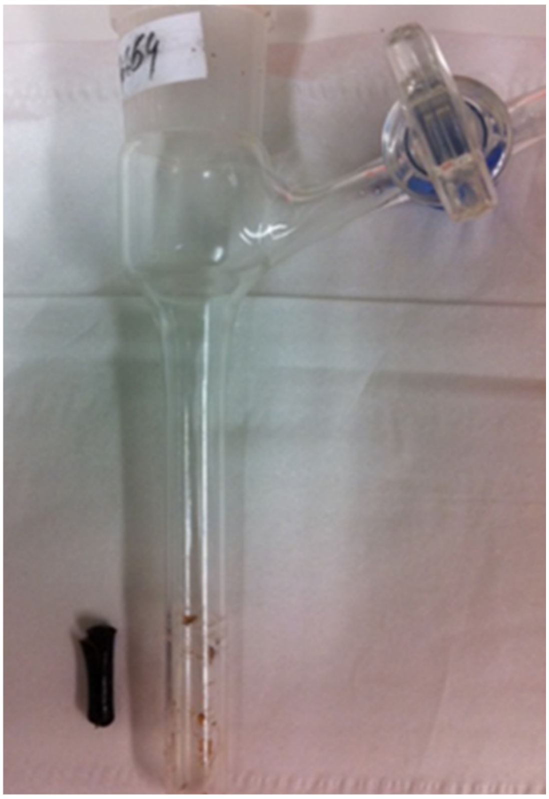

Figure S1. Photograph of the used Schlenk flask and the resulting monolithic Bet-PUR-2 material. The dark colour arises from impurities of the used Desmodur RE.

Porosity Analysis from CO_2 adsorption at 273 K

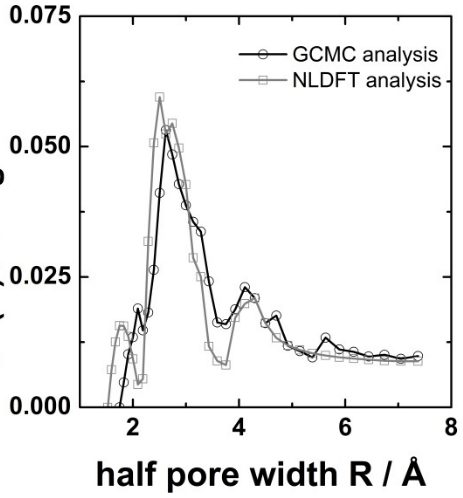

Figure S2. pore size distribution of Bet-PUR-1 as obtained from data evaluation based on GCMC or NLDFT models, CO_2 on carbon, slit pores, 273 K

FTIR

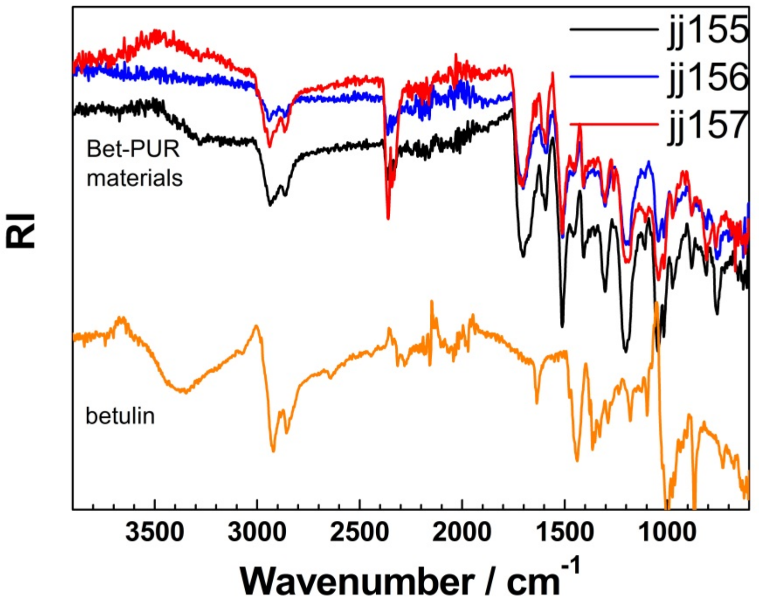

Figure S3. ATR-FTIR spectra of various batches of Bet-PUR-1 and betulin (orange).

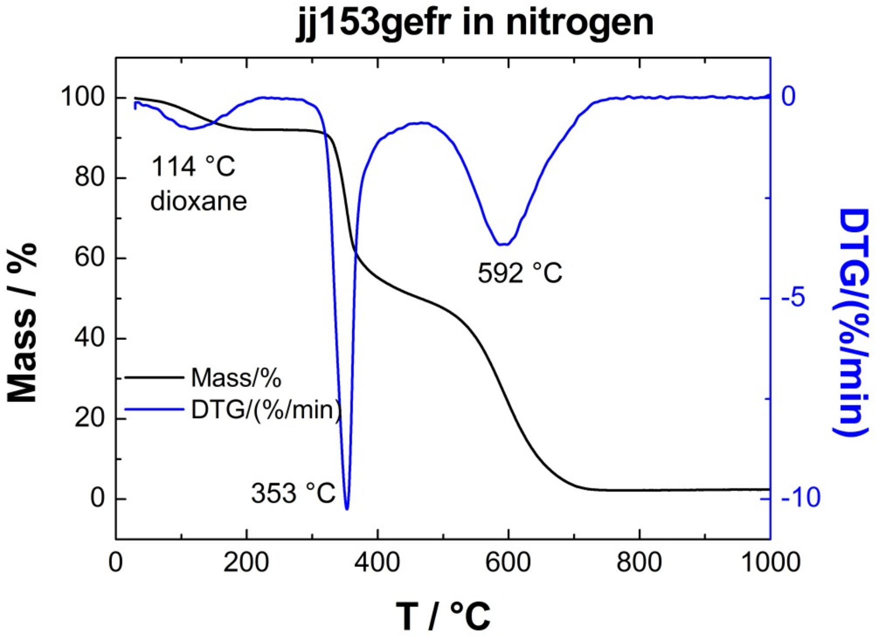

Figure S4. Typical TGA curve of Bet-PUR-1 (N_2 atmosphere, 10K/min)

Fitting parameters and IAST modeling

Experimental CO_2 adsorption isotherms of Bet-PUR-1 were fitted using a Langmuir model (single site or dual site), yielding an expression for the adsorbed amount q. N_2 adsorption isotherms were fitted using a single-site Langmuir approach, as dual-site fitting gave a similar result.

Single site Langmuir:

$$q = \frac{(q_i \cdot b_i \cdot p)}{(1 + b_i \cdot p)}$$

p: pressure of the bulk gas at equilibrium with the adsorbed phase, q: adsorbed amount, q_i: saturation capacities of component i, b_i affinity coefficients of component i

For curve fitting of the measured data points the following fit equation was used.

Using the measured data of CO_2 and N_2 isotherms at 273 or 283 K and the fit equation, the calculated isotherms were obtained:

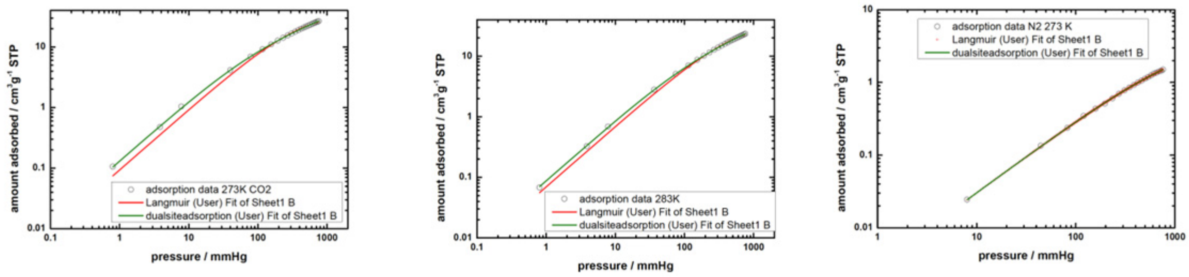

Figure S5. dual-site (green solid lines) and single site fits (red solid lines) of CO_2 at 273 K, 283K and N_2 at 273 K plotted together with the experimental data (open circles).

Table S1: single site fit parameters

	CO_2 (273 K)	CO_2 (283 K)	N_2 (273 K)
q	44.04848	41.01542	4.28742
b	0.00225	0.0017	7.11E-4
R^2	0.99841	0.9991	0.99971

Selectivity was calculated using the common IAST equation[1]:

$$\alpha(CO_2 / N_2) = \frac{x_{CO2} / x_{N2}}{y_{CO2} / y_{N2}}$$

where α is the selectivity, x the adsorbed amount and y represents the gas phase composition.

x_{CO2} was determined using a Newton-Raphson method implemented within a MatLab® (R2011a) script (see below)

(1) Myers, A. L.; Prausnitz, J. M. *AIChE Journal* **1965**, *11*, 121–127.

```
%Script for calculation of adsorbed amount of component A following IAST
%
%the script uses a Newton-Raphson methodology to calculate x
%input needed:  Langmuir parameter of pure component isotherms
%
%               ! pressure is set to mmHg (torr)!
%
%the script gives finally three plots of the spreading pressures for three
%different (arbitrarily choosen) values of x for illustration
%
%contact: jens.weber@mpikg.mpg.de

clear
q=input('input q_a: ');     %internal renaming to avoid use of subscripts)
a=input('input b_a: ');
u=input('input q_b: ');
b=input('input b_b: ');
y=input('input y_a: ');
p=10;

%create file;
S=input('input File Name (format ´filename.txt´): '); %please enter filename using the 'blabla.txt' notation
fid = fopen(S,'w','a');
fprintf(fid, 'parameters (for script using Newton-Raphson methodology):\n\n');
fprintf(fid, 'q_a and b_a:\n');
fprintf(fid, '%f %f\n\n', [q a]);
fprintf(fid, 'q_b and b_b:\n')
fprintf(fid, '%f %f\n\n', [u b]);
fprintf(fid, 'y_a =\n');
fprintf(fid, '%f\n\n', y);
fclose(fid)

for p=10:10:800
    syms x;
    f=(q*log(1+a*y*p/x))-(u*log(1+b*p*(1-y)/(1-x)));
    xRoot=0.999
    g = x-f/diff(f);                 %# Create a Newton-Raphson approximation function
    xRoot = subs(g,'x',xRoot)        %# Evaluate the function at the initial guess
    xNew = subs(g,'x',xRoot);        %# Refined guess
while abs(xNew-xRoot) > 1e-10         %# Loop while they differ by more than 1e-10
        xRoot = xNew;                %# Update the old guess
        xNew = subs(g,'x',xRoot);    %# Update the new guess
end
    xRoot = xNew;



p
xNew


fid = fopen(S,'a');                                     % open the file with permission to append
fprintf(fid, '%6.2f  %12.8f %6.2f\n', [p xNew i]);     % write values at end of file
fclose(fid);                                            % close the file

end

p=[1:800]
spreada = q*log(1+a*y*p/0.99)
spreadb = u*log(1+b*(1-y)*p/0.01);
plot (p,spreada,p,spreadb)
hlegl=legend('x adsorbed = 0.99')
pause
spreada = q*log(1+a*y*p/0.95)
spreadb = u*log(1+b*(1-y)*p/0.05);
plot (p,spreada,p,spreadb);
hlegl=legend('x adsorbed = 0.95')
pause
spreada = q*log(1+a*y*p/0.90)
spreadb = u*log(1+b*(1-y)*p/0.1);
plot (p,spreada,p,spreadb)
hlegl=legend('x adsorbed = 0.9')
```
